# Gradual scoliosis correction over time with shape-memory metal: a preliminary report of an experimental study

**DOI:** 10.1186/1748-7161-7-20

**Published:** 2012-11-05

**Authors:** José Miguel Sánchez Márquez, Francisco Javier Sánchez Pérez-Grueso, Nicomedes Fernández-Baíllo, Enrique Gil Garay

**Affiliations:** 1 Orthopedic Surgery Department, Spine Service, La Paz University Hospital, Madrid, Spain

## Background

Severe and progressive scoliosis is a complex three-dimensional spinal deformity that commonly requires treatment to address curve progression during growth. Standard treatment options for progressive scoliosis are essentially limited to bracing or surgery. Brace treatment is noninvasive and preserves growth; however it is only modestly successful in preventing curve progression and has a negative psychological impact [1-3] that may decrease patient compliance. Instead, surgical treatment with an instrumented spinal arthrodesis usually results in good deformity correction but has several risks. Those risks are associated to the invasiveness of spinal arthrodesis, the instantaneous correction of spinal deformity, and the altered biomechanics of the fused spine. Spinal fusion can have deleterious effects on subsequent development. Besides the known loss of motion and risk of adjacent segment disease with long-segment fusion, the loss of growth potential can lead to a significant decrease in trunk height and may negatively impact pulmonary development. Therefore, recent interest has been focused on new strategies for the effective surgical management of severe scoliosis in young children without the use of multisegmental spinal fusion. Fusionless scoliosis surgery provides theoretical advantages over traditional surgical arthrodesis, including the potential preservation of growth, motion and function of the spine [4].

In this way, several methods have been developed to treat scoliosis in young patients that allow the natural growth and elongation of the developing spine.

Nitinol is an alloy of almost equal atomic parts of Nickel and Titanium, it can be bent when cooled and then retake its original shape when heated. This characteristic, known as shape memory, and its pseudoelastic property, make nitinol an interesting material for deformity correction. Nitinol has different applications in the biomedical field [5,6]: orthodontic wires, cardiovascular surgery (cava vein filters or Self-Expanding stents), orthopedic surgery (spinal interbody spacers, shape-memory staples, plates) and the manufacture of surgical instruments.

The purpose of this study was twofold; firstly, to create a structural experimental scoliosis in a rat model by tethering sutures between the left scapula and pelvis; and secondly, to correct the scoliosis and resulting vertebral structural deformities over time, using a shape-memory alloy wire attached to the spine after the removal of the posterior tether. It was hypothesized that significant progressive scoliosis with structural vertebral body deformities would be achieved during the creation of an experimental scoliosis according to the Hueter-Volkmann Law. It was also hypothesized that a straight shape-memory alloy wire implantation in the spine would provide better gradual correction of the scoliosis and vertebral body deformities by maintaining steady corrective force loading on the still growing spine.

## Materials and methods

### Creation of the deformity

Twenty-three Sprague–Dawley male rats, between twenty and twenty-two days old, were used in this study, which was approved by our Institutional Animal Care and Use Committee. The number of rats was selected on the basis of a sample size and power analysis performed on the variance in spinal deformity creation and correction in five pilot animals. To create the scoliosis, we used a modification of the method described by Sarwark et al. [7].

After general anesthesia by intraperitoneal injection of a combination of ketamine, xylazine and atropine, dorsoventral and lateral radiographs of the spine were made. Next, the animals’ backs were shaved using an electric clipper and prepared with clorhexidine 4%. After antibiotic prophylaxis with an intramuscular injection of amoxicillin-clavulanic acid (150 mg/kg) and Oculos® tears lubricant ophthalmic ointment (Novartis Farma SA), a right kyphoscoliosis was induced by tethering the left scapula and the left pelvis. Two 1 cm incisions were made in the interscapular area and at the base of the tail. The scapula was exposed, grasped with forceps and a double loop of 0 polypropylene monofilament non-absorbable suture (Prolene®, Ethicon Inc, Johnson & Johnson; Somerville, NJ) was passed through the inferior angle of the left scapula; a third loop was made around the neck of the scapula. Both ends of the suture were passed subcutaneously to the second incision at the base of the tail. The end of the suture with the needle was passed around the left sacroiliac joint twice. Tightening the suture produced an instantaneous right-sided scoliosis. The wounds were then closed with 3/0 silk non-absorbable suture (Lab. Aragó®SL, Barcelona, Spain) and a posterior-anterior and lateral x-rays were taken. Rats were left in this position tethered for eight weeks.

Subcutaneous meloxican (3 mg/kg/day) was injected during the first two days post-op to control the pain.

Every two weeks the rats were sedated with the same intraperitoneal injection as before so that dorsoventral and lateral radiographs could be made. Both scoliotic and kyphotic curvatures were evaluated by the Cobb method.

### Correction of the deformity

After the 8-week tethering period, rats were randomized into treated (n = 9) and untreated groups (n = 9). All treated and untreated rats underwent cutting of the posterior tether under general anesthesia and ocular and antibiotic prophylaxis as described before. In the untreated group this was done through a 1 centimeter left posterior incision between both previous incisions. In the treated group, a posterior longitudinal midline incision was made to cut the tether, and subsequently a 0.5 mm x 0.5 mm Nitinol wire (NeoSentalloy® 0.018”x0.018” F100, GAC International Inc, Japan) was implanted on the spine along the concavity, from the upper end vertebra to the lower end vertebra, according to the preoperative x-ray. The 0.5 mm wire applies a 100 gram straightening force against the curved spine.

**Figure 1:**
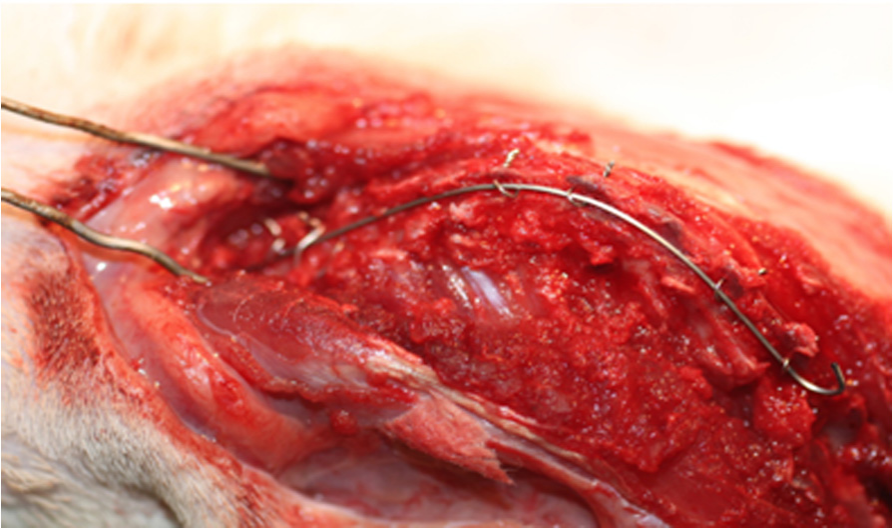
**Photograph of a rat with a Nitinol wire attached to the spine along the concavity.** Fixation was achieved by boring two holes through the spinous processes. Stainless-steel wires were passed through the holes and Nitinol wire rod was placed over the wires. The wires were then tightened at each point to ensure stabilization of the rod against the dorsal aspect of the spinal column. It is very important not to tighten the wires so much that necessary movement becomes impossible.

Fixation was achieved by boring two holes through the spinous processes using a microdrill at the lower two end vertebras and at the two vertebras in the apex. Making holes through the spinous processes in the upper end vertebras was impossible, given their thinness and resulting fragility during perforation. At the two upper end vertebras we made a loop with a 0.2 mm stainless-steel wire between the transverse and spinous process. Stainless-steel wires were passed through these holes and the Nitinol wire was placed over the wires. The wires were then tightened at each point to ensure stabilization of the rod against the dorsal aspect of the spinal column (Figure 1). It is very important not to tighten the wires too much so as to allow for necessary movement. The wound was sutured with 3/0 non-absorbable silk.

After surgery, the animals were given buprenorphine (0.05 mg/kg/day) subcutaneously for pain daily for the first three days, Lactated Ringer’s solution as needed, and Amoxicillin-Clavulanic acid (150 mg/kg/day) intramuscularly for three days to prevent wound and urinary tract infection. For the duration of the experiment, animals were observed daily by a veterinarian.

Radiographic imaging of the animals was performed immediately, 24 hours, 72 hours, 1 week and 2 weeks after surgery under intraperitoneal anesthesia as described above. Both scoliotic and kyphotic curves were evaluated.

Statistical analysis was performed using SAS 9.1, Enterprise Guide 3.0. To analyze the average change of scoliosis and kyphosis between groups over time, a mixed linear regression model was conducted. The fixed effect of time and intervention condition and their interaction were evaluated. Time was included as a repeated-effect covariate. A general covariance structure was used. Post hoc least-squares tests were conducted at each assessment point to identify any significant differences between groups. Ninety-five percent confidence intervals (CI) are shown for the main results. Two-sided tests were used and a p value of less than 0.05 was considered statistically significant.

## Results

### Creation of deformity

**Figure 2:**
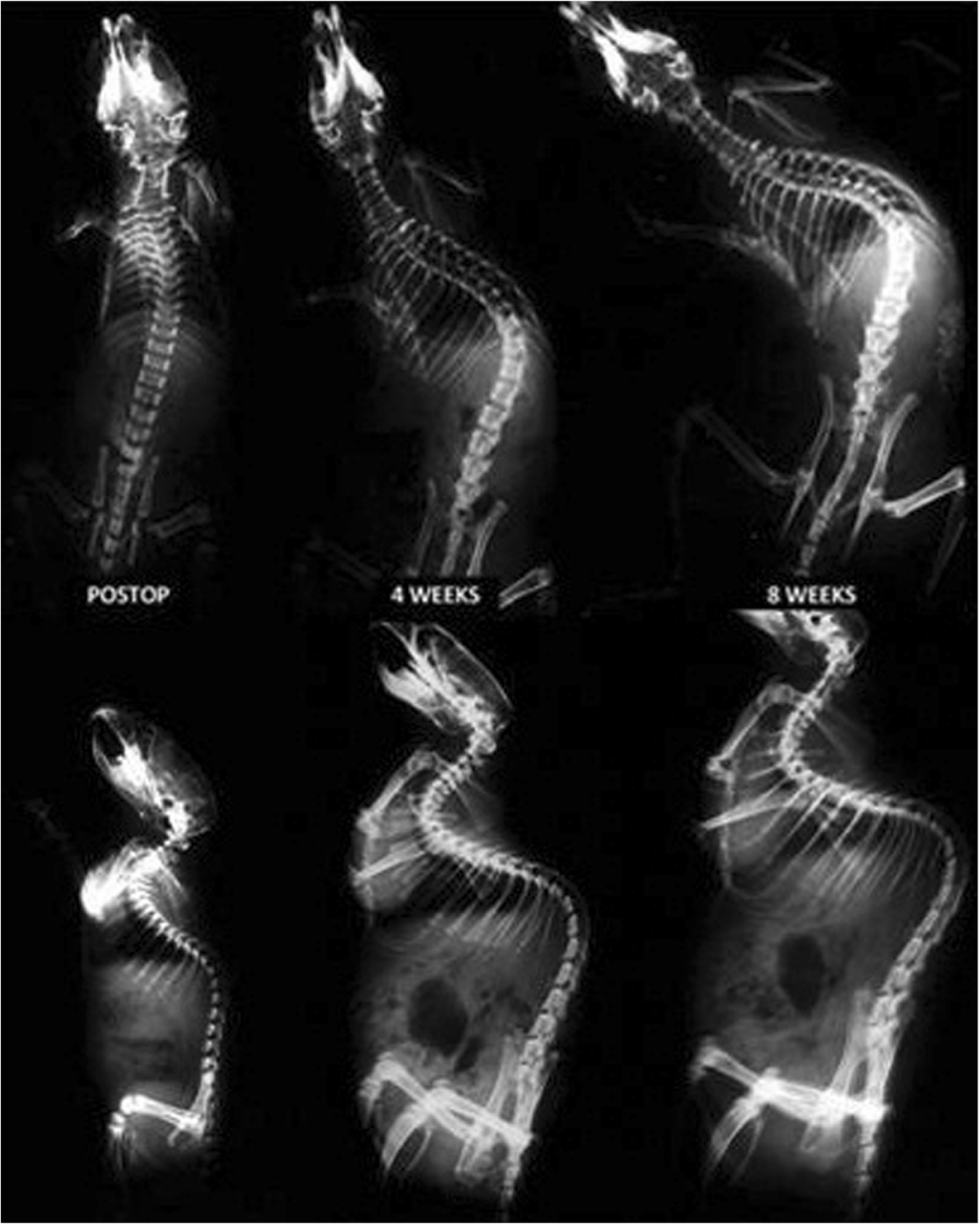
**Posterior-anterior and lateral radiographs of a rat during the tethering period, at immediate postop, at 4 and 8 weeks.** Progressive scoliosis and kyphosis are induced. Note the translation of the apical vertebra from the midline and rotation of the apical vertebral body.

During the tethering period, all rats except three developed a progressive, kyphoscoliotic curve of significant magnitude convex to the right in the thoracolumbar spine. In addition to the deformity in both coronal and sagittal planes, other characteristic radiographic features of idiopathic scoliosis were apparent. Radiographically, these include significant translation of the apical vertebra from the midline and rotation of the apical vertebral body (Figure 2).

The initial posterior-anterior x-ray showed a straight spine in every rat. Initial curves after posterior asymmetric tethering measured 35.5 degrees on average (CI:31.7-39.2), and progressed to 81.5 degrees (CI:78-85) on average at 8 weeks (Table 1). The average progression was statistically significant (Table 1).

**Table 1:** Scoliosis and kyphosis during the deformity induction

	**Preop**	**Post Inm**	**2 weeks**	**4 weeks**	**6 weeks**	**8 weeks**
SCOLIOSIS	0	35.5 (31.7-39.2)	46.2 (42.5-50)	66.6 (62.9-70.3)	72.7 (69-76.4)	81.5 (78-85)
		[22-57]	[30-60]	[48-90]	[59-90]	[60-95]
KYPHOSIS	47 (42.-51.5)	57.5 (53-62)	69.1 (64.6-73.6)	81.9 (77.4-86.4)	89.8 (85.3-94.3)	97.5 (94-101)
	[30-65]	[40-80]	[60-90]	[55-93]	[65-116]	[81-112]

Scoliosis and kyphosis mean value, confidence interval (in parentheses) and range: min-max [in brackets] during the deformity induction with a left asymmetric tethering.

In the lateral plane, all rats presented a normal sagittal profile, with 47 degrees (CI:42.5-51.5) of mean kyphosis before tethering. After tethering the mean kyphosis was 57.5 degrees (CI:53-62) and progressed to 97.5 degrees (CI:94-101) at 8 weeks. The kyphosis progression was statistically also significant.

Two rats died in the immediate postoperative period due to hypothermia and excessive bleeding. Three rats failed to develop scoliosis due to spontaneous tether released at some time after surgery in two cases, and a cut-out phenomenon in the scapula in one rat.

### Correction of deformity

During the treatment period, the treated rats demonstrated greater overall correction of kyphoscoliosis than the untreated rats. After randomization, the degree of kyphoscoliosis in both groups was homogeneous and there were no statistically significant differences in either plane in so far as Cobb angles between groups (p = 0.23 for scoliosis and p = 0.52 for kyphosis). In the treated group, the initial mean scoliosis measured 79.3 degrees (CI:73.9-84.7), and the final mean scoliosis measured 8.7 degrees (CI:3.3-14.1). We noted a progressive correction of the deformity over time (Table 2-A). In the untreated group, the initial mean scoliosis measured 84 degrees (CI:76.3-91.6) and the final scoliosis measured 54.3 degrees on average (CI:46.6-70). We noted an initial decrease in the Cobb angle after releasing the tether, but then the deformity remained stable and permanent over time. Differences between groups were statistically significant (p < 0.001).

**Table 2:** Scoliosis (A) and kyphosis (B) during de deformity correction


**A) Scoliosis correction**						
**SCOLIOSIS**	**Pre**	**Post Inm**	**24 h**	**72 h**	**1 week**	**2 weeks**
NiTi	79.3 (73.9-84.7)	51.6 (46.1-57)	32.4 (27-37.9)	27.9 (22.5-33.3)	21.5 (16.1-27)	8.7 (5.7-11.8)
	[70-89]	[34-63]	[27-40]	[20-37]	[17-33]	[4-16]
Control	84 (75.2-92.7)	60.2 (54.8-65.6)	58.6 (53.2-64)	57.2 (51.8-62.6)	55.7 (50.3-61.2)	54.3 (48.9-59.7)
	[60-95]	[48-77]	[45-75]	[40-73]	[40-70]	[40-70]
**B) Kyphosis correction**						
**KYPHOSIS**	**Pre**	**Post Inm**	**24 h**	**72 h**	**1 week**	**2 weeks**
NiTi	96.3 (90.4-102.2)	75.3 (69.4-81.2)	61.3 (55.4-67.2)	58.4 (52.5-64.3)	53.6 (47.7-59.5)	51.7 (45.8-57.6)
	[81-112]	[63-94]	[50-80]	[50-76]	[45-61]	[45-63]
Control	99 (93-104.9)	83 (77-88.9)	78.1 (72.2-84)	72.7 (66.8-78.7)	73.1 (67.2-79)	71.1 (65.2-77)
	[84-110]	[67-97]	[66-98]	[63-87]	[65-87]	[67-80]

Scoliosis and kyphosis mean value, confidence interval (in parentheses) and range [in brackets] during the deformity correction period, comparatively between treated and untreated groups.

The initial scoliosis correction after cutting the tether and implanting the NiTi wire differed between treated and untreated groups. The first group was corrected from 79.3 degrees to 51.5 degrees on average (-27.8 degrees) after removal of posterior tethering and the NiTi wire implantation; the untreated rats were corrected from 84 degrees to 60.2 degrees on average (-23.8) after removal of the posterior tether alone.

**Figure 3:**
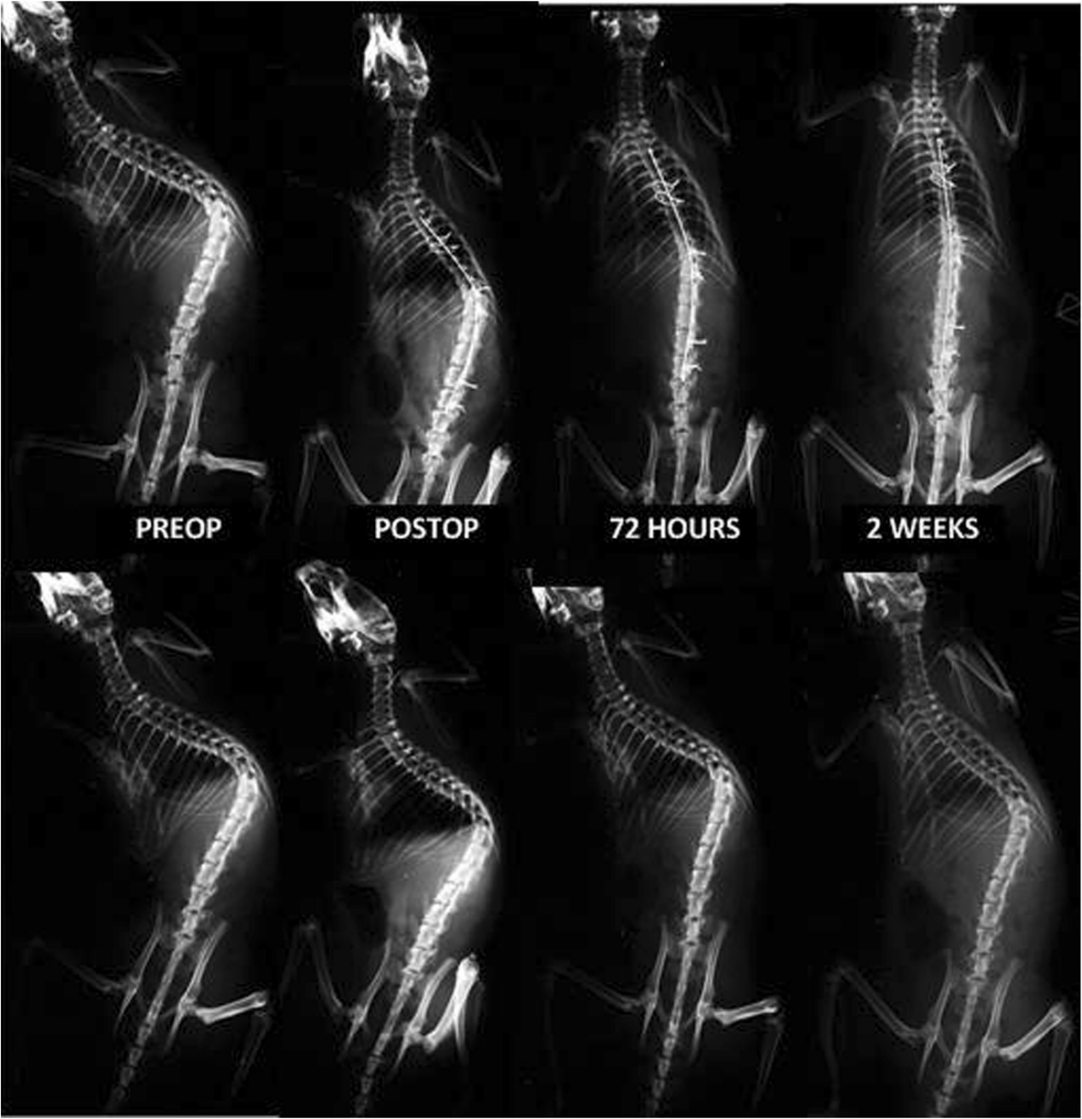
**Serial p-a radiographs during deformity correction, at the preoperative, at immediate postop, at 72 hours and 2 weeks (upper line: treated group; lower line: untreated group).** In the untreated group there was a slight decrease of deformity after tethering release but the deformity remained stable and permanent over time; in the treated group, gradual correction of the kyphoscoliosis was noted with the Cobb angle decreasing over time.

Over the course of the treatment period, treated rats showed a gradual correction of an additional 42.8 degrees in two weeks, whereas the untreated rats showed a mean correction of 6 degrees during that same time (Figure 3). The differences between the groups were statistically significant (p < 0.001).

**Figure 4:**
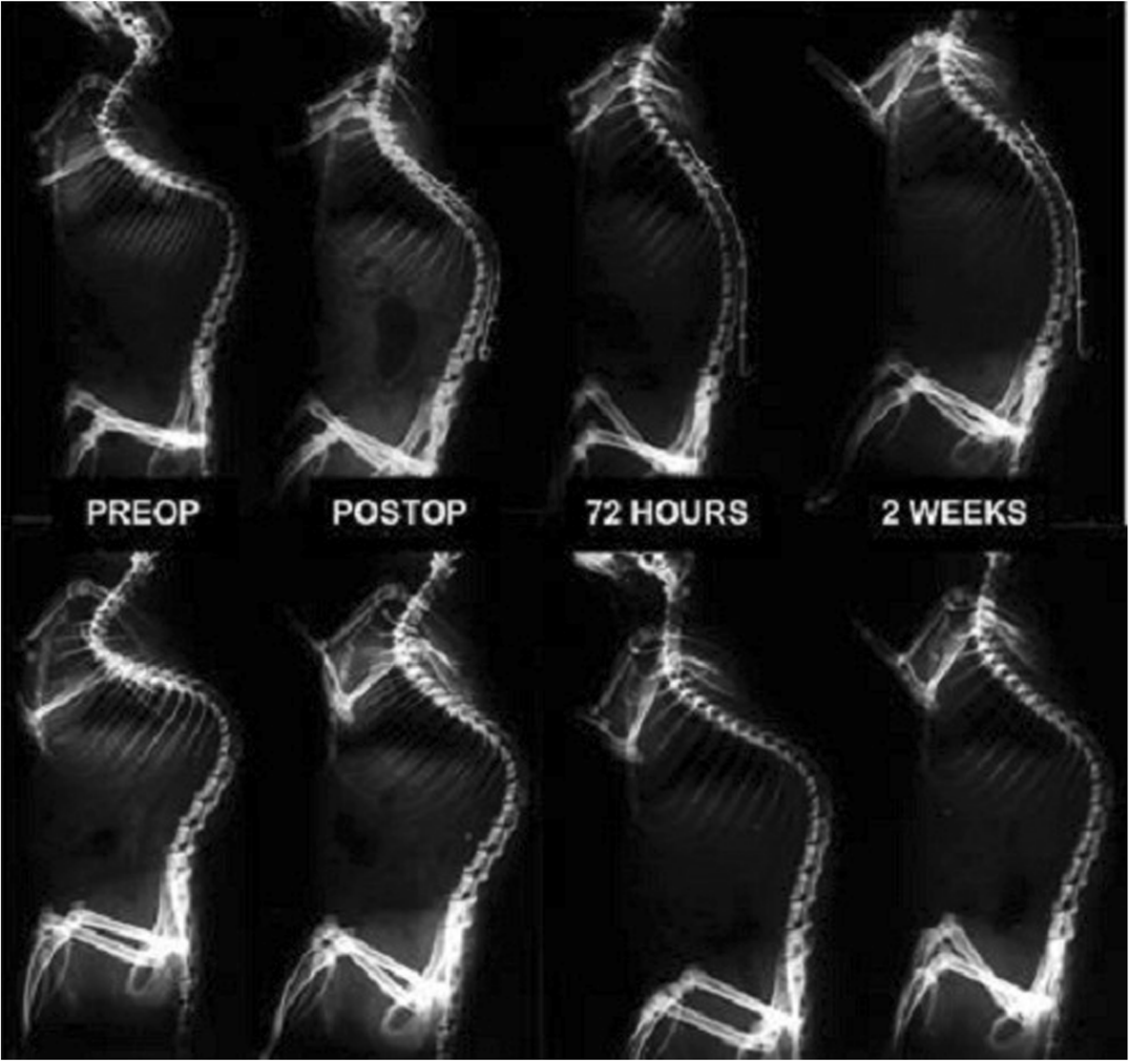
**Serial lateral radiographs during the deformity correction, at the preoperative, at immediate postop, at 72 hours and 2 weeks (upper line: treated group; lower line: untreated group).** Correction of kyphosis is not as apparent as that of scoliosis.

Examining the sagittal plane, treated rats demonstrated a greater correction of the kyphosis than untreated rats (Figure 4). The treated group went from 96.3 degrees on average (CI:90.4-102.2) at the beginning to 75.3 degrees on average (CI:69.4-81.2) after release of the tether and NiTi wire implantation, and to 51.7 degrees on average (CI:45.8-57.6) at two weeks after the corrective surgery. Untreated rats showed a lesser correction of their kyphosis going from 99 degrees (CI:93-104.9) to 83 degrees (CI:77-88.9) on average after tether release and to a mean Cobb angle of 71,1 degrees at two weeks (CI:65,2-77) (Table 2-B) . Differences were always statistically significant (p < 0.001).

On average, use of the NiTi wire decreased scoliosis by 70 degrees and kyphosis by 44 degrees at the end of two weeks in immature rats. Tether release (control group) decreased scoliosis by 30 degrees and kyphosis by 16°.

No rats died during the corrective surgical procedure. There was a caudal migration of NiTi wire in two rats, having lost the proximal anchorage; they required revision surgery to re-implant the wire through the upper anchorages at the upper thoracic spine. No differences were noted between rats that required revision surgery and the other rats.

## Discussion

In our daily practice, the surgical management of human scoliosis gets an instantaneous correction of the deformity by several maneuvers performed by the surgeon with the aid of spinal instrumentation. These maneuvers and instantaneous correction have several risks, including neurological injury due to spinal cord ischemia. Another drawback of conventional techniques is the spinal fusion, which can lead to a significant decrease in trunk height that will negatively impact pulmonary development. With shape-memory alloys and their ability to recover a previously defined shape when subjected to heat, a new form of gradual scoliosis correction has become possible: a force-driven correction, in which a shape-memory NiTi wire acts as a correction element over time.

The alloy used in this study was Nickel-Titanium in straight orthodontic wires with a square cross-section (0.5mmx0.5 mm). Initially, the temperature of the wire is below the transition temperature (32 Celsius degrees), in the martensite phase. In this phase, the wire has a very low yield strength, can be deformed quite easily and can be fixed to the scoliotic spine using a relatively easy procedure. When the wire is heated above 32 Celsius degrees, it moves into the austenite phase, and will gradually regain its original shape and rigidity. If this shape recovery is prevented, the wire generates considerable shape recovery stresses.

Several recent studies [8-10] performed by a single research group have evaluated the efficacy of anterior thoracic vertebral stapling with shape-memory alloys to correct a scoliosis and vertebral wedge deformities previously created in goats. The results note a poor to fair ability to correct these deformities.

Recently, Wang et al. [11,12] reported their results with the use of a shape memory alloy rod, as a temporary intraoperative tool for deformity correction that was replaced in the same surgical procedure by rigid rods at the end of correction. They noted that the temporary use of shape memory rods reduced the operational time, blood loss and achieved better three-dimensional correction than the use of only a standard rigid rod.

However, the current approach to scoliosis treatment is the use of permanent rods despite the inherent drawbacks of instantaneous correction.

In this experimental study, the shape memory wire induced a gradual correction of the scoliosis over time, non-instantaneously, without fusion, because it maintained a steady straightening force. This strategy has the theoretical advantage of avoiding the risk of neurological injury associated with instantaneous correction, and should preserve the spinal growth. The main difference of our study with others is that correction was not only produced during the surgery and rod heating, but continued over time after the surgery.

It should be kept in mind that this animal study was performed on the previously healthy spine of young animals, after inducing scoliotic deformation. Another point is that it should be remembered that the results of scoliosis creation and correction in an animal study are not always extrapolatable, to humans, but these results do offer translational possibilities.

Several groups have also investigated NiTi for scoliosis treatment. To our knowledge, the first attempt was made by investigators in Germany [13]. A NiTi memory wire was fixed to the convex side of 8 plastic model vertebrae in a curved shape. On being heated, the wire shortened and the model assumed a straight shape. Veldhuizen et al. [14] designed a device consisting of a shape-memory rod with a programmed scoliotic curve attached to a cadaver spine with hooks and pedicle screws. Heating the rod to 50°C produced a scoliotic curve with a Cobb angle of about 45°.

Sanders et al. [15] used six goats with an experimental scoliosis that was straightened with a 6 mm section nitinol rod. The rod was transformed and the scoliosis corrected. The curves averaged 41 degrees before instrumentation, 33 degrees after instrumentation and 11 degrees after rod transformation. A similar study was conducted on monkeys [16], in which two shape-memory metal rods were attached to the spine with transspinous wires. A good correction of the previously induced deformity was achieved instantaneously, but no extra and gradual correction was obtained after surgery.

In six immature pigs, Wever et al. [17] used an originally curved square NiTi rod (6.35x6.35 mm) to progressively bend the spine using pedicular screws at T12, L2 and L4. They obtained an induced scoliosis with an approximate Cobb angle of 40 degrees Cobb angle in the immediate postoperative radiographs (the same as the original rod curve) that remained constant during follow-up.

Curvature or its correction have been achieved instantaneously in experimental studies employing the NiTi shape memory alloy rods, but they have not reported progressive changes after the surgery itself. Rather than a robust rod, we have used a resilient wire that applies a light, but constant, tensile support to a growing spine. This gentle force, in combination with the inherent visco-elastic properties of the spine, would correct the deformity and theoretically avoid the neurological injuries associated with sudden spinal manipulation.

## Conclusions

In summary, given the results of this study, shape-memory alloys can be expected to be suitable for a new scoliosis management strategy of gradual postoperative correction over time, due to the interaction of corrective forces from NiTi wires and the viscous properties of the spine. Further studies are needed to optimize the implant, maximize the effectiveness of various surgical strategies and identify the appropriate indications for treatment.

## Competing interests

The authors declare that they have no financial or non-financial competing interests.

## Authors’ contributions

JMSM, FJSPG, NFB and EGG participated in the conception and design of the study. JMSM developed the experimental surgical procedures, made the x-rays and collected the data. All authors have been involved in analysis and interpretation of data. All authors have been involved in drafting the manuscript and have given the final approval of the version to be published.
